# Secondary decompressive craniectomy after severe traumatic brain injury: a retrospective cohort study

**DOI:** 10.3389/fneur.2025.1641639

**Published:** 2025-09-16

**Authors:** Wenwen Che, Cheng’ao Feng, Xiaomei Xu, Qiuyan Shen, Jianwei Rao, Yuhai Wang

**Affiliations:** ^1^Wuxi Medical College of Jiangnan University, Wuxi, Jiangsu, China; ^2^Department of Neurosurgery, No. 904 Hospital of the Joint Logistics Support Force of the Chinese People's Liberation Army, Wuxi, Jiangsu, China

**Keywords:** traumatic brain injury, decompressive craniectomy, secondary surgery, IPTW, prognosis

## Abstract

**Background:**

Severe traumatic brain injury (sTBI) often results in malignant intracranial hypertension, requiring decompressive craniectomy (DC). Although guidelines emphasize adequate decompression, craniectomy size is often individualized in practice. Secondary DC may be necessary when initial decompression is insufficient. This study investigated the risk factors and outcomes associated with secondary DC in sTBI patients.

**Methods:**

We conducted a retrospective cohort study of 101 sTBI patients who underwent DC between 2021 and 2023. Patients were divided into two groups: those receiving only primary DC (*N* = 85) and those requiring secondary DC (*N* = 16). Logistic regression identified predictors of secondary DC, while inverse probability of treatment weighting (IPTW) was applied to adjust for confounders.

**Results:**

Of the 101 patients who underwent DC, 85 received primary DC alone, while 16 required secondary DC. Patients in the secondary DC group had lower admission GCS scores (6.06 ± 2.95 vs. 7.88 ± 3.48, *p* = 0.038), higher preoperative ICP (45.4 ± 18.5 mmHg vs. 30.3 ± 16.2 mmHg, *p* = 0.007), and smaller initial craniectomy areas (110 ± 31.5 cm^2^ vs. 133 ± 51.4 cm^2^, *p* = 0.024). Multivariable regression identified preoperative ICP (OR 1.06, 95% CI 1.00–1.11, *p* = 0.038) and craniectomy area (OR 0.98, 95% CI 0.96–1.00, *p* = 0.037) as independent predictors of secondary DC. IPTW-adjusted analyses showed no significant differences in functional outcomes or complication rates.

**Conclusion:**

Secondary DC may serve as an effective salvage intervention in sTBI patients with refractory intracranial hypertension following primary DC. Although these patients present with more severe initial conditions, secondary DC did not increase the risk of complications or lead to poorer outcomes. Ensuring adequate decompression during the initial surgery may help reduce the need for secondary intervention.

## Introduction

Traumatic brain injury (TBI) is a pressing global public health and socioeconomic concern, affecting approximately 70 million individuals annually (1, 2). While the majority of TBI cases are classified as mild, about 20% involve severe injuries (1, 3, 4). Severe TBI (sTBI) is distinguished by extensive intracranial damage, significant disruption of neurological function, and a high mortality rate, ranging between 7 and 39% (3, 5, 6).

The management of sTBI is a formidable challenge in neurocritical care, necessitating prompt stabilization of hemodynamic and ventilatory parameters, continuous intracranial pressure (ICP) monitoring, and a systematic, tier-based therapeutic approach to mitigate intracranial hypertension and prevent secondary brain injury (7). Elevated ICP is a central therapeutic focus in sTBI management, as unchecked pressure increases within the rigid cranial vault can lead to the reduction of cerebral perfusion pressure, where sustained malignant intracranial hypertension can result in cerebral ischemia, brain herniation, and potentially death (8).

Despite ongoing controversy, decompressive craniectomy (DC) remains a pivotal surgical intervention in sTBI, demonstrating clear benefits in reducing intracranial pressure, preventing brain herniation, and improving survival in selected patients (9, 10). By removing a portion of the skull, DC creates space for the swollen brain to expand, alleviating the pressure within the rigid cranial vault and reducing the risk of secondary complications (11, 12). Although standard recommendations exist regarding the extent of decompression, the size of the initial craniectomy is often individualized in clinical practice, based on patient-specific anatomy, intraoperative findings, and surgeon judgment. However, in some cases, initial DC may fail to achieve sufficient ICP control, leading to ongoing intracranial hypertension, brain herniation, and potentially irreversible neurological damage (13). These scenarios pose major therapeutic challenges, as conventional medical measures—including osmotic agents (e.g., mannitol), sedation and analgesia, controlled hyperventilation, and mild hypothermia—are often insufficient to reverse the effects of sustained ICP elevation. If unrelieved, elevated ICP can compromise cerebral perfusion, result in ischemia, and markedly increase the risk of fatal brain herniation (14).

To bridge this therapeutic gap, secondary decompressive craniectomy (secondary DC) has been employed as a salvage intervention in patients with refractory intracranial hypertension after initial surgery, which creates additional space within the rigid cranial vault to accommodate brain swelling and prevent secondary complications. By enlarging the craniectomy area, this approach enhances the effectiveness of ICP reduction, ultimately improving cerebral perfusion and reducing the likelihood of adverse outcomes. Despite its theoretical advantages, the efficacy and safety of secondary DC remains uncertain, and the procedure is not without significant risks. The expanded surgical scope introduces greater technical challenges, which may increase the likelihood of complications such as infection, cerebrospinal fluid leaks, or long-term neurological sequelae. Here, this study aims to explore the clinical profiles and outcomes of patients undergoing secondary DC, providing valuable insights into its effectiveness and safety in the context of refractory ICP.

## Methods

### Data source and population

**Figure 1:**
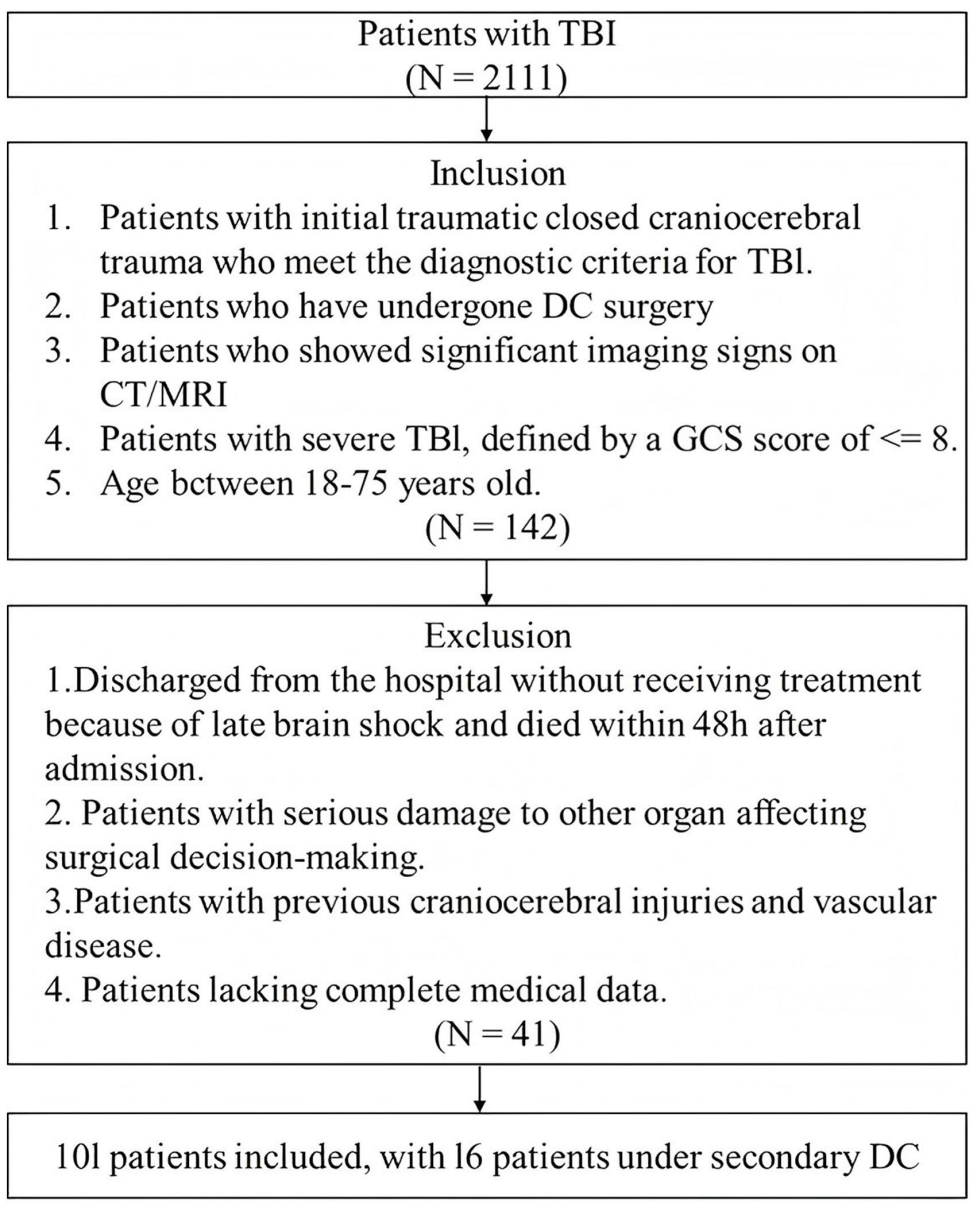
Flowchart of the study.

This retrospective cohort study was conducted at No. 940 Hospital of the PLA Joint Logistics Support Force, including data collected between Jan. 2021 and Dec. 2023. Patients aged 18–75 years, with initial acute closed severe TBI, defined by a GCS score of ≤8 or a coma duration of more than 12 h, and presenting with clear positive neurological signs and vital sign disturbances were eligible for inclusion, if they underwent DC. Patients were excluded if they were discharged without receiving treatment, died within 48 h of admission due to late-stage brain herniation without undergoing surgery, had a history of traumatic brain injury or cerebrovascular disease, or presented with severe comorbid organ dysfunction that impacted surgical decision-making. Those were also excluded if comprehensive medical records or radiological imaging (MRI or CT scans) were unavailable, ensuring the reliability of clinical and imaging data. The inclusion and exclusion criteria are summarized in a flowchart (Figure 1).

Data were collected through a retrospective chart review using a standardized data abstraction form. Key clinical and radiological variables were independently extracted from electronic medical records by two investigators. The extracted datasets were then compared for consistency, and any discrepancies or ambiguous entries were resolved through discussion and consensus; if necessary, a third senior investigator was consulted. This process was implemented to enhance data reliability and minimize errors inherent to retrospective reviews.

The study protocol was reviewed and approved by the Ethics Committee of the Hospital (20240401). Given the retrospective nature of the study, informed consent was waived in accordance with ethical standards. All procedures adhered to the principles of the Declaration of Helsinki, ensuring patient confidentiality and ethical research conduct.

### Outcomes and candidate predictors

The primary outcome of this study was defined as the risk of requiring secondary DC, evaluated by analyzing demographic, and clinical factors that may contribute to the need for this intervention. Candidate predictor variables were identified through a comprehensive review of clinical and epidemiological literature. Age and gender (*male* and *female*) were included as key sociodemographic factors. Clinical variables encompassed a broad spectrum of characteristics associated with TBI severity and surgical management. These included: mechanism of injury (*simple falls*, *vehicle accidents*, and *falls from height*), GCS at baseline, Marshall CT classification at baseline (*I* to *VI*), ICP before and after primary DC, presence of brain herniation or systemic shock, and surgical and procedural variables such as airway reconstruction, hematoma evacuation, ICP monitoring, and drainage (all categorized as *yes* or *no*). Additionally, the timing and extent of primary DC (time to surgery and craniectomy area), as well as medical history (presence of hypertension, diabetes, and previous surgeries) were included. Laboratory tests conducted both before and after primary DC were also included in the analysis, encompassing prothrombin time (PT), activated partial thromboplastin time (APTT), thrombin time (TT), fibrinogen (FIB), D-dimer, and prothrombin activity (PTA). Hematological parameters comprised platelet count (PLT), hemoglobin (HB), red blood cell count (RBC), white blood cell count (WBC), and lymphocyte count (LYMPH), albumin (ALB), immunoglobulin (IG), lactate dehydrogenase (LDH), alkaline phosphatase (ALP), and the platelet-to-lymphocyte ratio (PLR). These variables were selected because they are well-recognized predictors of mortality and poor outcome following severe TBI, as previously demonstrated in retrospective analyses of head-injured patients (15).

The secondary outcomes focused on the prognosis following secondary DC, encompassing GCS and the Glasgow Outcome Scale–Extended (GOSE) at discharge and 6 months post-surgery, which provided insights into neurological recovery and overall functional status. Additionally, secondary outcomes included the incidence of post-surgical complications, such as secondary seizures, infarction, infections, cognitive dysfunction, hydrocephalus, and cerebral infarction.

### Surgical procedures

All decompressive craniectomies were performed by six attending neurosurgeons working in three fixed two-person teams who alternated emergency duty shifts. While a general institutional guideline for decompressive craniectomy was available, it did not mandate a fixed bone flap size. The final extent of decompression was determined intraoperatively based on the severity of brain swelling, radiological findings, and the surgical team’s judgment. A review of surgical records revealed no clear pattern suggesting that smaller craniectomy sizes or secondary decompressions were concentrated among specific surgeons or teams.

Of note, in this study, initial DC was defined as the first decompressive craniectomy performed for severe TBI. Secondary DC was defined as any subsequent decompression, including ipsilateral revision/extension or contralateral procedures.

### Statistical analysis

Continuous data were analyzed using the independent *t*-test, while categorical variables were assessed using the chi-square test, continuity-corrected chi-square test, or Fisher’s exact test, as appropriate. Descriptive statistics for continuous variables were reported as mean (standard deviation [SD]) or median (interquartile range [IQR]), depending on data distribution, and categorical variables were expressed as frequencies and percentages.

Univariate and multivariate logistic regression analyses were conducted to identify risk factors associated with the requirement for extended decompressive craniectomy (secondary DC). Initially, candidate predictors were evaluated using univariate logistic regression, with predictors meeting a significance threshold of *p* < 0.1 selected for inclusion in the multivariate logistic regression model. Adjusted odds ratios (ORs), accounting for age and sex, were calculated to provide a robust estimation of the associated risk factors.

For the analysis of prognosis associated with secondary DC, to minimize confounding bias and balance baseline characteristics between groups, inverse probability of treatment weighting (IPTW) was applied (16). Predictors were first refined using stepwise multivariate logistic regression. The final logistic regression model was refitted using backward elimination guided by the Akaike information criterion (AIC) to derive the minimal adequate model for predicting the need for secondary DC (17). Based on the results of the logistic regression analysis, propensity scores were calculated to represent the probability of requiring secondary DC given the observed baseline characteristics. These propensity scores were then used to create a weighted pseudo-population with balanced covariates across groups. Baseline characteristics between groups were compared using both conventional statistical tests (*t*-test or chi-square test, as appropriate) and standardized mean differences (SMDs). *p* values were considered the primary criterion for assessing group comparability, with *p* < 0.05 indicating statistical significance. SMDs were reported in parallel as a descriptive measure of imbalance, with values <0.25 generally regarded as negligible.

All analyses were performed using R statistic software (version 4.3.1). Statistical significance was defined as a two-tailed *p*-values of less than 0.05, unless otherwise specified.

## Results

### Population characteristics

Between 2021 and 2023, a total of 101 patients underwent DC for severe TBI. Of these, 85 patients received only primary DC, while 16 required secondary DC. Baseline demographic and clinical characteristics are presented in Table 1. The mean age of the cohort was 51.84 years, with males comprising the majority (*N* = 66, 65.3%). Vehicle accidents were the predominant mechanism of injury, accounting for 73 cases (72.3%), followed by other causes. The mean GCS score at admission was 7.59 (SD 3.46) and radiological assessments revealed that most patients were classified as Marshall CT grades III (*N* = 35, 34.7%) and VI (*N* = 45, 44.6%). The average ICP measured 33.3 mmHg (SD 17.64) prior to primary DC, which reduced to 15.75 mmHg (SD 15.75) after the procedure. The mean DC area was approximately 129.50 cm^2^ (SD 49.40). Procedural interventions included airway reconstruction in 9 patients (8.9%), hematoma evacuation in 73 patients (72.3%), ICP monitoring in 62 patients (61.4%), and drainage in 4 patients (4.0%).

**Table 1:** Summary characteristics of patients with initial acute closed severe TBI at baseline.

Variables (%/mean [SD])	Overall	Primary craniectomy	Secondary craniectomy	*p*-value
No.	101	85 (100)	16 (100)	–
Demographic information				
Age	51.84 (15.01)	51.6 (15.9)	53.3 (8.84)	0.537
Gender				0.98
Male	66 (65.3)	55 (64.7)	11 (68.8)	
Female	35 (34.7)	30 (35.3)	5 (31.2)	
Time to primary surgery	34.26 (73.77)	32.7 (58.4)	42.6 (131)	0.77
Clinical information				
Reason				0.664
Ground-level falls or simple falls	11 (10.9)	10 (11.8)	1 (6.25)	
Motor vehicle accidents	73 (72.3)	62 (72.9)	11 (68.8)	
Fall from a height	17 (16.8)	13 (15.3)	4 (25.0)	
Herniation				0.385
No	82 (71.3)	62 (72.9)	10 (62.5)	
Yes	29 (28.7)	23 (27.1)	6 (37.5)	
Shock				0.241
No	95 (94.1)	81 (95.3)	14 (87.5)	
Yes	6 (5.9)	4 (4.71)	2 (12.5)	
Marshall CT				0.062
I	0 (0.0)	0 (0.0)	0 (0.0)	
II	15 (14.9)	14 (16.5)	1 (6.25)	
III	35 (34.7)	32 (37.6)	3 (18.8)	
IV	3 (3.0)	3 (3.53)	0 (0.00)	
V	3 (3.0)	1 (1.18)	2 (12.5)	
VI	45 (44.6)	35 (41.2)	10 (62.5)	
GCS at baseline	7.59 (3.46)	7.88 (3.48)	6.06 (2.95)	0.038
Intervention				
Airway reconstruction				0.005
No	92 (90.1)	81 (95.3)	11 (68.8)	
Yes	9 (8.9)	4 (4.71)	5 (31.2)	
Hematoma evacuation				0.037
No	28 (17.7)	27 (31.8)	1 (6.25)	
Yes	73 (72.3)	58 (68.2)	15 (93.8)	
ICP monitoring				0.134
No	39 (38.6)	36 (42.4)	3 (18.8)	
Yes	62 (61.4)	49 (57.6)	13 (81.2)	
Drainage procedure				1
No	97 (96.0)	81 (95.3)	16 (100)	
Yes	4 (4.0)	4 (4.71)	0 (0.00)	
Preoperative ICP	33.30 (17.64)	30.3 (16.2)	45.4 (18.5)	0.007
Postoperative ICP	15.75 (6.60)	15.4 (6.37)	17.3 (7.46)	0.347
Bone flap area	129.50 (49.40)	133 (51.4)	110 (31.5)	0.024
Type of DC				
Ipsilateral			12 (75)	
Contralateral			4 (25)	
Complications				
Hypertension				0.302
No	101 (80.2)	70 (82.4)	11 (68.8)	
Yes	20 (19.8)	15 (17.6)	5 (31.2)	
Diabetes				1
No	96 (95.0)	80 (94.1)	16 (100)	
Yes	5 (5.0)	5 (5.88)	0 (0.00)	
Surgical history				1
No	96 (95.0)	80 (94.1)	16 (100)	
Yes	5 (5.0)	5 (5.88)	0 (0.00)	

GCS, Glasgow coma scale; ICP, Intracranial pressure.

Among the reasons for requiring secondary DC, intracranial hypertension was the most common indication. Brain herniation syndromes (*N* = 5) represented the primary clinical manifestation while radiological findings included midline shift (*N* = 6), delayed hematoma formation (*N* = 4), intracerebral hematomas (*N* = 2), and brainstem compression (*N* = 1), all indicative of progressive intracranial pressure elevation and the need for surgical decompression.

### Risk factors for secondary decompressive craniectomy

Patients in the secondary DC group presented with significantly worse GCS scores at admission (6.06 ± 2.95 vs. 7.88 ± 3.48, *p* = 0.038). Advanced Marshall CT stages were more frequently observed in the secondary DC group, although this difference did not reach statistical significance (*p* = 0.062). Preoperative ICP was notably higher in patients requiring secondary DC (45.4 ± 18.5 mmHg vs. 30.3 ± 16.2 mmHg, *p* = 0.007) but postoperative ICP levels were comparable between the two groups. Regarding surgical factors, the secondary DC group was characterized by a smaller decompression area during the initial DC procedure (110 ± 31.5 cm^2^ vs. 133 ± 51.4 cm^2^, *p* = 0.024). Furthermore, patients in the secondary DC group were more likely to undergo additional procedures, such as airway reconstruction and hematoma evacuation (both *p* < 0.05).

In terms of laboratory findings (Table 2), the secondary DC group exhibited higher thrombin time levels (19.5 ± 4.06 vs. 17.2 ± 2.27, *p* = 0.081) and significantly lower fibrinogen levels (2.61 ± 1.38 vs. 1.78 ± 1.02, *p* = 0.02) following primary DC, while no significant differences were observed in other laboratory parameters.

**Table 2:** Summary of laboratory test of patients with traumatic brain injury after first surgery.

Variables	Primary craniectomy	Secondary craniectomy	*p*-value
No.	85	16	
Preoperative lab test			
PT	13.0 (1.78)	13.5 (3.22)	0.666
APTT	29.2 (5.62)	42.6 (42.8)	0.324
TT	19.4 (6.27)	31.6 (42.6)	0.364
FIB	1.73 (0.72)	1.57 (0.75)	0.525
D-D	33.1 (33.8)	46.9 (39.1)	0.292
PTA	89.9 (20.7)	93.3 (32.2)	0.741
PLT	184 (70.0)	174 (65.2)	0.602
Hb	125 (24.2)	132 (21.0)	0.297
RBC	4.08 (0.76)	4.25 (0.70)	0.39
WBC	16.1 (6.95)	14.2 (4.62)	0.166
LYMPH	2.47 (2.02)	3.09 (2.13)	0.293
Postoperative lab test^1^			
PT	13.6 (1.80)	14.2 (2.45)	0.383
APTT	32.5 (6.76)	41.9 (33.0)	0.325
TT	17.2 (2.27)	19.5 (4.06)	0.081
FIB	2.61 (1.38)	1.78 (1.02)	0.02
D-D	14.1 (19.7)	22.7 (25.0)	0.259
PTA	82.7 (22.7)	79.6 (20.5)	0.633
PLT	120 (52.9)	101 (45.6)	0.149
Hb	98.2 (19.7)	93.5 (21.2)	0.423
RBC	3.21 (0.69)	3.03 (0.71)	0.349
WBC	12.8 (3.90)	12.3 (3.72)	0.649
LYMPH	0.86 (0.71)	0.80 (0.48)	0.719
CV^2^	0.36 (1.46)	0.17 (0.07)	0.263
SD	37.9 (6.93)	39.4 (6.80)	0.44
ALB^2^	30.8 (7.67)	31.9 (5.77)	0.573
IG	21.7 (21.1)	33.1 (51.3)	0.425
LDH	242 (93.6)	243 (84.2)	0.952
ALP	49.0 (39.7)	45.8 (19.0)	0.643
PLR	188 (125)	176 (145)	0.776

PT, prothrombin time; APTT, activated partial thromboplastin time; TT, thrombin time; FIB, fibrinogen; D-dimer, D-dimer; PTA, prothrombin activity; PLT, platelet count; HB, hemoglobin; RBC, red blood cell count; WBC, white blood cell count; LYMPH, lymphocyte count; PLR, platelet-to-lymphocyte ratio; ALB, albumin; IG, immunoglobulin; LDH, lactate dehydrogenase; ALP, alkaline phosphatase.

1. “Postoperative” refers to the period following primary decompressive craniectomy (DC).

2. Variables such as preoperative albumin (ALB) and central venous pressure (CV) were not included in the analysis due to missing data exceeding 20%.

Univariate logistic regression identified several factors associated with an increased risk of requiring secondary DC (Table 3). Advanced Marshall CT stage (OR 28.00, 95% CI 1.21–648.85), lower GCS scores at admission (OR 0.84, 95% CI 0.69–1.01), higher preoperative ICP (OR 1.05, 95% CI 1.02–1.09), smaller decompression area during surgery (OR 0.99, 95% CI 0.98–1.00), receipt of airway reconstruction (OR 9.20, 95% CI 2.14–39.55), hematoma evacuation (OR 6.98, 95% CI 0.88–55.62), and ICP monitoring (OR 3.18, 95% CI 0.84–12.00) were all identified as potential risk factors.

**Table 3:** Logistic regression analysis of factors associated with secondary decompressive craniectomy.

Variables	Univariable regression		Multivariable regression with age and sex adjustment^2^		Final multivariable regression (AIC-selected)^3^	
	OR	*p*-value	OR	*p*-value	OR	*p*-value
Age	1.01 (0.97–1.05)	0.668	1.01 (0.97–1.05)	0.563		
Gender						
Male	Ref	–	Ref	–		
Female	0.83 (0.26–2.62)	0.755	0.82 (0.26–2.58)	0.73		
Time	1 (1–1.01)	0.625	1 (1–1.01)	0.647		
Reason						
Ground-level falls or simple falls	Ref	–	Ref	–		
Motor vehicle accidents	1.77 (0.21–15.28)	0.602	1.79 (0.21–15.52)	0.595		
Fall from a height	3.08 (0.3–31.97)	0.347	3.11 (0.29–33.11)	0.347		
Herniation						
No	Ref	–	Ref	–		
Yes	1.62 (0.53–4.95)	0.4	1.62 (0.53–4.99)	0.397		
Shock						
No	Ref	–	Ref	–		
Yes	2.89 (0.48–17.32)	0.245	2.97 (0.49–17.91)	0.235		
Airway						
No	Ref	–	Ref	–	Ref	–
Yes	9.2 (2.14–39.55)	0.003	10.66 (2.27–50.15)	0.003	3.95 (0.57–27.46)	0.165
Marshall CT						
II	Ref	–	Ref	–	Ref	–
III	1.31 (0.13–13.74)	0.82	1.38 (0.12–15.65)	0.793	0.69 (0.04–13.33)	0.809
IV	–	0.992	–	0.992	–	0.993
V	28 (1.21–648.85)	0.038	31.84 (1.25–813.49)	0.036	25.6 (0.34–1,909.64)	0.140
VI	4 (0.47–34.24)	0.206	4.14 (0.43–40.12)	0.221	1.03 (0.06–17.87)	0.982
Hypertension						
No	Ref	–	Ref	–		
Yes	2.12 (0.64–7.01)	0.218	2.22 (0.58–8.44)	0.243		
Diabetes						
No	Ref	–	Ref	–		
Yes	–	0.993	–	0.993		
Surgery						
No	Ref	–	Ref	–		
Yes	–	0.993	–	0.993		
Haematoma removal						
No	Ref	–	Ref	–	Ref	–
Yes	6.98 (0.88–55.62)	0.066	6.85 (0.85–55.33)	0.071	8.66 (0.44–171.7)	0.157
ICP monitoring						
No	Ref	–	Ref	–	Ref	–
Yes	3.18 (0.84–12)	0.087	3.53 (0.91–13.66)	0.068	0.7 (0.11–4.36)	0.705
ICP before surgery	1.05 (1.02–1.09)	0.004	1.05 (1.02–1.09)	0.005	1.06 (1–1.11)	0.038
ICP after surgery	1.05 (0.96–1.14)	0.29	1.05 (0.96–1.14)	0.267		
Area	0.99 (0.98–1)	0.092	0.99 (0.97–1)	0.08	0.98 (0.96–1)	0.037
GCS	0.84 (0.69–1.01)	0.06	0.84 (0.69–1.01)	0.06	1.03 (0.78–1.36)	0.843
Mannitol						
No	Ref	–	Ref	–		
Yes	0.42 (0.14–1.27)	0.125	0.42 (0.14–1.3)	0.133		
Albumin						
No	Ref	–	Ref	–		
Yes	0.6 (0.2–1.78)	0.355	0.6 (0.2–1.78)	0.356		
Before surgery						
PT						
Normal	Ref	–	Ref	–		
Low	0.25 (0.04–1.65)	0.148	0.25 (0.04–1.7)	0.158		
High	0.3 (0.03–2.65)	0.278	0.3 (0.03–2.68)	0.28		
APTT						
Normal	Ref	–	Ref	–		
Low	0.39 (0.09–1.69)	0.207	0.38 (0.09–1.7)	0.207		
High	2 (0.32–12.33)	0.455	1.94 (0.3–12.44)	0.483		
FIB						
Normal	Ref	–	Ref	–		
Low	1.63 (0.54–4.88)	0.384	1.66 (0.54–5.06)	0.375		
PTA						
Normal	Ref	–	Ref	–		
Low	1.16 (0.3–4.53)	0.826	1.22 (0.31–4.81)	0.782		
WBC						
Normal	Ref	–	Ref	–		
High	0.57 (0.17–1.86)	0.35	0.59 (0.18–1.99)	0.398		
LYMPH						
Normal	Ref	–	Ref	–		
Low	–	0.993	–	0.993		
After Surgery						
PT						
Normal	Ref	–	Ref	–		
Low	0.3 (0.02–3.6)	0.339	0.3 (0.02–3.71)	0.35		
High	0.55 (0.04–7.09)	0.643	0.55 (0.04–7.21)	0.652		
APTT						
Normal	Ref	–	Ref	–		
Low	0.56 (0.06–5.61)	0.624	0.56 (0.06–5.71)	0.628		
High	1.41 (0.13–15.27)	0.776	1.39 (0.13–15.11)	0.787		
FIB						
Normal	Ref	–	Ref	–		
Low	0.51 (0.17–1.52)	0.225	0.51 (0.16–1.59)	0.245		
High	–	0.991	–	0.991		
PTA						
Normal	Ref	–	Ref	–		
Low	0.78 (0.26–2.36)	0.654	0.83 (0.26–2.68)	0.76		
PLT						
Normal	Ref	.	Ref	.		
Low	0.86 (0.29–2.52)	0.779	0.85 (0.29–2.5)	0.765		
WBC_01_Category						
Normal	Ref	–	Ref	–		
Low	0.63 (0.2–2.05)	0.446	0.66 (0.2–2.18)	0.498		
LYMPH						
Normal	Ref	–	Ref	–		
Low	–	0.995	–	0.995		
High	–	0.993	–	0.993		
CV^1^						
Normal	Ref	–	Ref	–		
High	0.43 (0.13–1.45)	0.174	0.43 (0.13–1.45)	0.175		
SD						
Normal	Ref	–	Ref	–		
Low	–	0.994	–	0.994		
ALB^1^						
Normal	Ref	–	Ref	–		
Low	0.62 (0.2–1.94)	0.41	0.63 (0.2–2)	0.435		
LDH						
Normal	Ref	–	Ref	–		
High	0.98 (0.31–3.09)	0.968	1 (0.31–3.17)	0.996		
ALP						
Normal	Ref	–	Ref	–		
High	2.08 (0.62–7)	0.236	2.12 (0.62–7.27)	0.233		
Normal	–	0.993	–	0.993		
PLR						
Normal	Ref	–	Ref	–		
High	–	0.991	–	0.991		
Normal	–	0.991	–	0.991		

GCS, Glasgow coma scale; ICP, Intracranial Pressure; PT, prothrombin time; APTT, activated partial thromboplastin time; TT, thrombin time; FIB, fibrinogen; D-dimer, D-dimer; PTA, prothrombin activity; PLT, platelet count; HB, hemoglobin; RBC, red blood cell count; WBC, white blood cell count; LYMPH, lymphocyte count; PLR, platelet-to-lymphocyte ratio; ALB, albumin; IG, immunoglobulin; LDH, lactate dehydrogenase; ALP, alkaline phosphatase.

1. Variables such as preoperative albumin (ALB) and central venous pressure (CV) were not included in the analysis due to missing data exceeding 20%.

2. These multivariable regression models were adjusted for age and sex.

3. In the final multivariable model, all predictors with *p* < 0.1 were retained, and the model was selected according to the AIC criterion.

Multivariable logistic regression (selected according to the AIC) further confirmed that higher preoperative ICP and a smaller decompression area were significant predictors of secondary DC.

### Prognostic and complications following secondary decompressive craniectomy

There were no significant differences in the incidence of complications between the secondary DC and primary DC groups within 6 months after surgery (Table 5). The most common complications observed in both groups included seizures, infections, and cognitive dysfunction.

**Table 4:** Summary of baseline of patients with severe traumatic brain injury after first surgery after IPTW.

Variables	Primary craniectomy	Secondary craniectomy	*p*-value
No. (%)	81.73	63.97	–
Age (mean [SD])	52.10 (14.38)	51.93 (6.56)	0.944
Gender			0.725
Male	46.9 (57.4)	41.6 (65.0)	
Female	34.8 (42.6)	22.4 (35.0)	
Time to primary surgery (mean [SD])	28.82 (51.76)	27.87 (72.85)	0.954
Reason			0.4
Ground-level falls or simple falls	8.5 (10.4)	1.2 (1.9)	
Motor vehicle accidents	59.6 (72.9)	46.8 (73.2)	
Fall from a height	13.6 (16.7)	16.0 (24.9)	
Herniation			0.139
No	49.9 (61.1)	52.4 (81.8)	
Yes	31.8 (38.9)	11.6 (18.2)	
Shock			0.828
No	66.3 (93.4)	60.6 (94.7)	
Yes	5.4 (6.6)	3.4 (5.3)	
Airway reconstruction			0.852
No	68.8 (84.3)	55.3 (86.4)	
Yes	12.9 (15.7)	8.7 (13.6)	
Hematoma evacuation			0.736
No	26.0 (31.9)	15.5 (24.3)	
Yes	55.7 (68.1)	48.5 (75.7)	
ICP monitoring			0.894
No	18.4 (22.5)	13.1 (20.4)	
Yes	63.3 (77.5)	50.9 (79.6)	
Drainage procedure			0.11
No	77.0 (94.3)	64.0 (100)	
Yes	4.7 (5.7)	0.0 (0.0)	
Marshall CT			< 0.001
II	10.3 (12.6)	1.5 (2.3)	
III	35.8 (43.8)	5.5 (8.6)	
IV	2.1 (2.5)	0.0 (0.0)	
V	1.1 (1.3)	17.1 (26.7)	
VI	32.5 (39.8)	39.9 (62.5)	
GCS (mean [SD])	7.17 (3.53)	6.25 (2.35)	0.282

**Table 5:** Summary of complications of patients with severe traumatic brain injury after surgery.

Variables	Before IPTW			After IPTW		
	Primary decompressive craniectomy	Secondary decompressive craniectomy	*p*-value	Primary decompressive craniectomy	Secondary decompressive craniectomy	*p*-value
No.	85	16		81.73	63.97	
Seizures	14 (16.5)	1 (6.25)	0.454	9.9 (12.1)	15.5 (24.2)	0.455
Infarction	3 (3.53)	0 (0.00)	>0.99	1.3 (1.5)	0.0 (0.0)	0.391
Infection	14 (16.5)	1 (6.25)	0.454	10.8 (13.2)	1.7 (2.7)	0.104
Cognitive	5 (5.88)	2 (12.5)	0.306	4.5 (5.5)	11.8 (18.4)	0.184
Hydrocephalus	3 (3.53)	1 (6.25)	0.504	2.2 (2.7)	1.5 (2.4)	0.924
Cerebral Infarction	2 (2.35)	0 (0.00)	>0.99	1.0 (1.3)	0.0 (0.0)	0.394
Discharge GOSE Score	5.51 (1.99)	4.62 (2.33)	0.173	4.82 (2.27)	4.94 (2.41)	0.91
6-Month GOSE Score	3.16 (1.21)	2.44 (1.15)	0.032	2.85 (1.27)	2.89 (1.13)	0.925
Discharge GCS Score	6.38 (3.31)	4.56 (2.28)	0.012	5.42 (2.77)	4.74 (2.11)	0.366

GCS, Glasgow coma scale.

However, patients who underwent secondary DC demonstrated significantly lower GCS scores at discharge compared to those who received primary DC (4.56 ± 2.28 vs. 6.38 ± 3.31, *p* = 0.012). Although no significant differences in the GOSE scores were observed at discharge, the secondary DC group exhibited significantly lower GOSE scores at 6 months post-surgery (2.44 ± 1.15 vs. 3.16 ± 1.21, *p* = 0.032).

Based on the results of the logistic regression analysis, propensity scores were computed using a logistic regression model with the following covariates: time to surgery, airway reconstruction, hematoma evacuation, primary craniectomy area and ICP before primary DC. After adjusting for confounding factors using IPTW (Table 4, Supplementary Table 1), no significant differences were observed between the secondary DC and primary DC groups with respect to either complications or prognosis (All *p* > 0.05).

**Figure 2:**
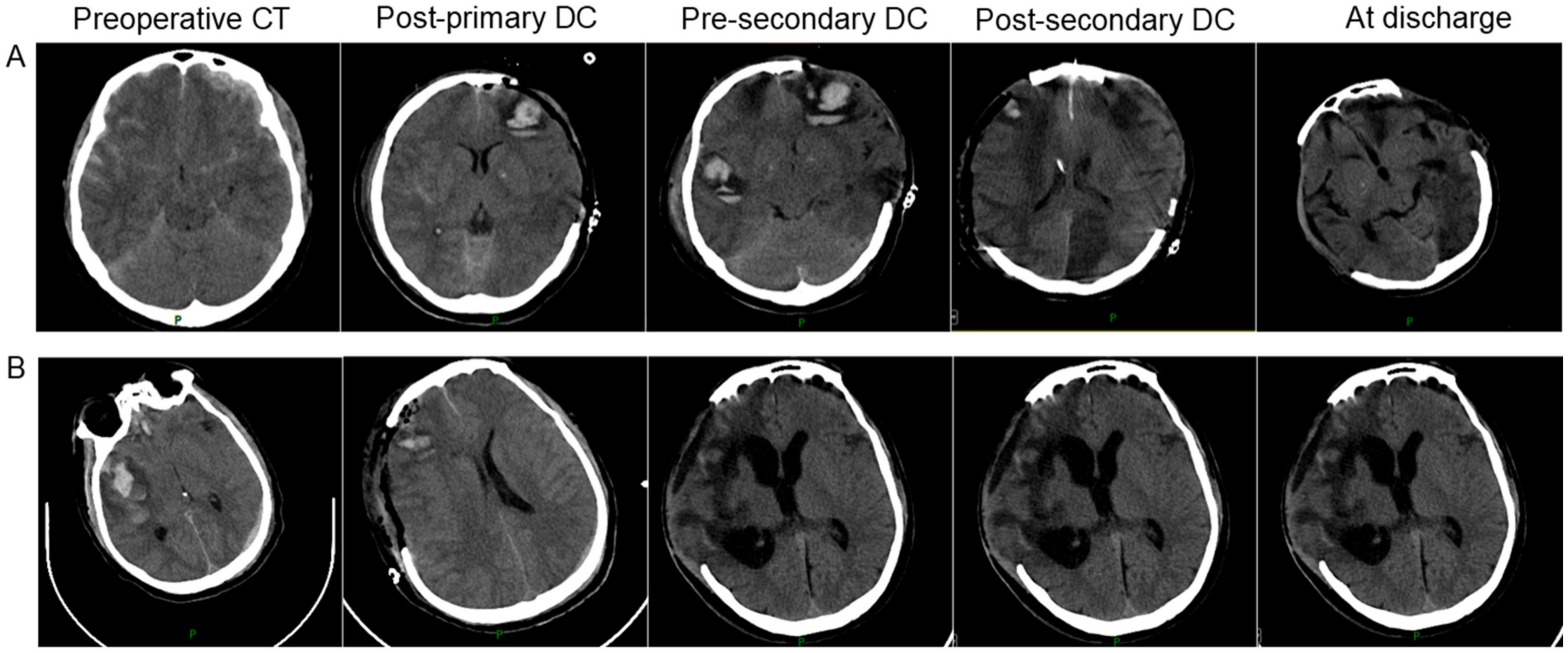
Representative cases of secondary decompressive craniectomy in severe traumatic brain injury. **(A)** An adult patient underwent right-sided primary DC (9 × 9 cm) for bilateral frontal contusions and left occipital epidural hematoma. Due to progressive cerebral edema and ICP elevation (40 mmHg), an ipsilateral secondary DC (12 × 15 cm) was performed. GOSE at 6 months was 3; complications included pneumonia, post-traumatic epilepsy, and hydrocephalus. **(B)** An adult patient underwent left-sided primary DC (10 × 11 cm) for a large subdural hematoma. Persistent elevated ICP (34 mmHg) and new hematoma formation led to a contralateral (right-sided) secondary DC (12 × 15 cm). GOSE at 6 months was 4; complications included pneumonia and deep vein thrombosis.

In summary, to further illustrate our findings, we have selected two representative cases for presentation, as shown in Figure 2.

## Discussion

This retrospective cohort study provides a comprehensive understanding of the clinical characteristics, outcomes, and risks associated with secondary decompressive craniectomy in severe TBI. Our findings highlight significant differences in baseline characteristics between patients who underwent secondary DC and those who received only primary DC. Specifically, patients in the secondary DC group presented with more severe initial conditions, including significantly lower GCS scores at admission, higher preoperative ICP, and more advanced Marshall CT stages. Logistic regression analysis further identified higher preoperative ICP, and smaller decompression areas during primary DC were significant predictors associated with the need for secondary DC. Notably, no significant differences were observed between the two groups in terms of complications or prognosis.

This study underscores the critical relationship between higher preoperative intracranial pressure and the need for secondary DC, as well as the influence of smaller decompression areas during the primary DC procedure. ICP is primarily regulated by the volume of three components: brain tissue, cerebrospinal fluid. Following traumatic brain injury, the intracranial volume may increase due to hemorrhage, cerebral edema, or hydrocephalus, leading to a dangerous rise in ICP. The rigid structure of the skull exacerbates this problem, as the brain has limited space to accommodate swelling (18). Accordingly, if ICP remains high despite initial medical management, decompressive craniectomy is typically performed to alleviate the pressure. DC has long been used as a surgical intervention to manage refractory ICP by removing a portion of the skull, thereby providing additional space for the swollen brain and reducing pressure. The physiological goal of DC is to reduce ICP to a level that allows for improved cerebral blood flow and prevents brain herniation. DC can be particularly effective in cases of elevated ICP not responding to medical management, but the timing and extent of decompression remain key factors in determining its success (18, 19).

Guidelines from major TBI management consortia, such as the Brain Trauma Foundation, recommend early and adequate decompression to prevent irreversible brain injury (20, 21). However, there is still no universal agreement on the optimal timing for primary DC. Most guidelines suggest that DC should be performed when ICP exceeds 20–25 mmHg for extended periods, despite maximal medical management. By removing a substantial portion of the skull, this procedure effectively reduces ICP and mitigates the risk of secondary brain injury. However, despite its benefits, excessive decompression may occur in some patients, leading to a range of postoperative complications. These complications can include syndrome of the trephined, paradoxical herniation, cerebrospinal fluid disturbances, and delayed brain shifts, all of which may negatively impact neurological recovery. Therefore, the optimal extent and location of decompression plays a critical role in surgical outcomes. Typical decompression areas include the frontal, temporal, and parietal regions, with the extent of the craniectomy depending on the severity of the injury and the patient’s clinical status. That is to say, there is no universally defined “ideal” decompression area, which can lead to situations where ICP remains elevated even after the initial DC (22, 23). This insufficiency in primary decompression may subsequently result in the need for secondary decompressive craniectomy to further alleviate intracranial pressure. This highlights the importance of ensuring an appropriately sized decompression area during the primary procedure to reduce the likelihood of requiring further interventions. Future research should focus on defining the optimal decompression area based on injury severity and individual patient factors to minimize the need for secondary surgical interventions.

However, it is noteworthy that after rigorous adjustment, we found no significant differences in prognosis or complications between patients who underwent only primary decompressive craniectomy and those who required secondary DC. This may suggest that secondary DC does not result in an increased risk of complications or secondary injury compared to primary DC. Therefore, when primary decompression fails to adequately control intracranial pressure, performing secondary DC may not exacerbate risks and could be a reasonable intervention to prevent further neurological deterioration (24). However, repeated surgeries can increase both the economic and physiological burden on patients. The need for additional procedures not only prolongs the hospital stay and escalates medical costs but also exposes patients to extended recovery times (25). Therefore, while secondary DC may be necessary in some cases to prevent further neurological deterioration, it emphasizes the importance of ensuring that the initial decompressive surgery is adequately performed. Optimizing surgical decision-making to reduce the need for subsequent interventions could help mitigate the physical and financial burdens on patients and the healthcare system. Further research should focus on refining guidelines for primary DC (26).

Beyond its acute role in ICP control, our findings also relate to the ongoing debate on optimal craniectomy size and its long-term implications. Our observation that smaller initial craniectomy areas were associated with a higher likelihood of requiring secondary decompression underscores the importance of ensuring adequate decompressive size. This finding dovetails with a broader debate in neurotrauma: how large should a decompressive craniectomy be? Too small a bone flap may fail to adequately control ICP, necessitating revision as seen in our study, whereas excessively large defects may carry their own risks. For example, large craniectomy defects have been linked to the syndrome of the trephined, a delayed complication characterized by neurological deterioration due to loss of cranial integrity, which typically improves after cranioplasty (27). Furthermore, craniectomy size can influence reconstructive outcomes; large or bilateral defects may increase the complexity and complication rates of cranioplasty. A recent multicenter study identified defect size as a factor in predicting cranioplasty complications (28). Taken together, these considerations highlight the need to balance acute ICP control with long-term risks when determining the extent of decompression.

According to the BTF guidelines, DC is recommended as a second-tier therapy for refractory intracranial hypertension in severe TBI, with the primary goal of improving survival, although its impact on long-term functional outcome remains complex. Our findings support these recommendations in several respects. Secondary DC effectively controlled intracranial pressure without significantly worsening functional outcomes, consistent with the notion that timely decompression can be life-saving without invariably resulting in poor neurological recovery, as also reflected in the RESCUEicp trial. In addition, our observation that inadequate initial decompression often necessitated secondary DC underscores the guideline emphasis on performing a sufficiently large bone flap removal (e.g., a wide fronto-temporo-parietal craniectomy of at least 12 cm diameter reaching the skull base and midline when appropriate).

Building on these guideline principles, our study also provides actionable recommendations for neurosurgeons. First, ensuring adequate initial decompression is critical; maximizing the extent of the bone flap and performing dural expansion when indicated may help reduce the need for subsequent surgery. Second, patients presenting with markedly elevated ICP or diffuse/bilateral injury patterns should be recognized as high risk and considered for a more aggressive primary approach, including larger or bilateral DC where appropriate. Third, developing institutional protocols that standardize minimum craniectomy size and indications for bilateral decompression may reduce variability among surgeons and improve outcomes (29). Finally, even after adequate DC, vigilant postoperative ICP monitoring remains essential, with timely consideration of secondary DC in cases of refractory hypertension before irreversible injury occurs.

Taken together, these findings align with BTF guideline recommendations while also adding nuance: in our cohort, secondary DC did not significantly increase the risk of severe disability compared with primary DC alone, in contrast to earlier concerns raised by DECRA and other trials. This suggests that, with careful patient selection and appropriate surgical technique, secondary DC can be considered a viable salvage strategy consistent with current guidelines, while emphasizing that the best opportunity to prevent secondary intervention lies in performing an extensive and technically adequate primary decompression.

Several limitations of this study must be acknowledged. Firstly, as a retrospective cohort study, it is inherently limited by biases associated with observational data, such as selection bias and information bias. The decision to perform secondary DC was not randomized but based on clinical judgment, which introduces the potential for unmeasured confounders that may affect both the surgical decision and the outcomes. Therefore, prospective studies are needed to mitigate these limitations and provide stronger evidence for the effectiveness of secondary DC. Secondly, the retrospective single-center design and relatively small sample size, particularly the secondary DC group of 16 patients, limit the statistical power of our analysis and raise the possibility of Type II errors, that is, failing to detect true differences or associations due to insufficient sample size. For example, although no significant differences in functional outcomes were observed between groups after IPTW adjustment, small to moderate effects may have gone undetected. The wide confidence intervals for some outcome measures further reflect this uncertainty. Accordingly, we have interpreted the negative findings with caution and recommend that future studies with larger multicenter cohorts be conducted to validate our results. Additionally, this study was conducted at a single center, which may limit the generalizability of the findings to other institutions with different patient populations, surgical practices, and postoperative care protocols. Multi-center studies would be invaluable in improving the external validity of the results and offering a broader perspective on the outcomes associated with secondary DC. Furthermore, due to the retrospective nature of this study, surgeon-specific data on craniectomy size were not consistently documented, preventing a formal stratified analysis of inter-surgeon variability. Although our review did not indicate that smaller craniectomy sizes or secondary decompressions were concentrated within specific surgical teams (Supplementary Table 2), we acknowledge this as a limitation. Future prospective studies with standardized documentation of surgical decision-making would better clarify the influence of individual surgical practice on outcomes. While the study assessed functional outcomes at 6 months post-surgery, a longer follow-up period would be beneficial to fully evaluate the long-term cognitive, psychological, and quality-of-life outcomes in patients who undergo secondary DC. These additional insights would be crucial for understanding the lasting effects of this intervention and improving long-term patient care. Lastly, although baseline characteristics were generally comparable between groups after weighting, some imbalances remained. For example, except for CT, all baseline variables showed *p* values > 0.05, suggesting no statistically significant differences. However, the SMD for CT exceeded the conventional threshold, indicating residual imbalance. This may be explained by the clinical context and the nature of our dataset: CT is closely linked to treatment selection and disease status in practice, which makes it inherently more difficult to achieve balance through weighting. Moreover, given the limited sample size and heterogeneity of the study population, subtle imbalances are expected and may persist despite statistical adjustment.

Conclusively, this study provides a comprehensive analysis of the clinical characteristics, outcomes, and risk factors associated with extended decompressive craniectomy in patients with severe traumatic brain injury. The findings indicate that higher ICP and smaller decompression areas during the initial surgery may contribute to the need for secondary DC. These factors underscore the critical importance of achieving adequate decompression in the early stages of treatment to prevent the need for further surgical interventions. Additionally, the study found no significant differences in complications or prognosis between the secondary DC and primary DC groups, suggesting that the potential of secondary DC as a life-saving procedure for managing refractory ICP, without an additional risk of postoperative complications compared to primary DC.

## Data Availability

The raw data supporting the conclusions of this article will be made available by the authors without undue reservation.
