# Supplementary material for: Secondary decompressive craniectomy after severe traumatic brain injury: a retrospective cohort study
Source: Front Neurol. 2025 Sep 16;16:1641639. doi: 10.3389/fneur.2025.1641639 (PMC12479320; doi:10.3389/fneur.2025.1641639)
Supplement: Supplementary file 1 [file Table_1.DOCX]

**Supplementary Table 1. Standardized Mean Differences (SMD) Before and After Weighting for Baseline Covariates**

| **Covariate** | **SMD before IPTW** | **SMD after IPTW** |
| --- | --- | --- |
| Age | 0.1 | 0.02 |
| Sex | 0.16 | 0.16 |
| Time to primary surgery | 0.10 | 0.01 |
| Reason | 0.20 | 0.40 |
| Herniation | 0.17 | 0.47 |
| Shock | 0.22 | 0.05 |
| Airway construction | 0.68 | 0.06 |
| Hematoma evacuation | 0.85 | 0.17 |
| ICP monitoring | 0.19 | 0.05 |
| Drainage procedure | 0.37 | 0.35 |
| Marshall CT | 0.86 | 1.33 |
| GCS | 0.51 | 0.31 |

**Supplementary Table 2. Comparison of craniectomy size and secondary decompression across surgical teams.**

| Team | No. (%) | Mean area (cm^2^, mean +SD) | *P* value | Secondary DC, No. (%) | *P* value |
| --- | --- | --- | --- | --- | --- |
| A | 24 (23.8) | 126.5 (46.0) |  | 3(12.5) |  |
| B | 38 (37.6) | 131.2 (50.5) |  | 6 (15.8) |  |
| C | 39 (38.6) | 128.0 (48.0) |  | 6 (15.4) |  |
| Total | 101 | 129.5 (49.4) | 0.73 | 16 | 0.92 |
